# Myeloid-Derived Suppressor Cells: A New and Pivotal Player in Colorectal Cancer Progression

**DOI:** 10.3389/fonc.2020.610104

**Published:** 2020-12-15

**Authors:** Kai Yin, Xueli Xia, Ke Rui, Tingting Wang, Shengjun Wang

**Affiliations:** ^1^ Department of General Surgery, Affiliated Hospital of Jiangsu University, Zhenjiang, China; ^2^ Department of Immunology, Jiangsu Key Laboratory of Laboratory Medicine, School of Medicine, Jiangsu University, Zhenjiang, China; ^3^ Department of Laboratory Medicine, Affiliated Hospital of Jiangsu University, Zhenjiang, China; ^4^ Department of Laboratory Medicine, Affiliated Wuxi People’s Hospital of Nanjing Medical University, Wuxi Children’s Hospital, Wuxi, China

**Keywords:** myeloid-derived suppressor cells, colorectal cancer, cancer immunology, colorectal cancer immunotherapy, tumor microenvironment

## Abstract

Colorectal cancer (CRC) remains a devastating human malignancy with poor prognosis. Of the various factors, immune evasion mechanisms play pivotal roles in CRC progression and impede the effects of cancer therapy. Myeloid-derived suppressor cells (MDSCs) constitute an immature population of myeloid cells that are typical during tumor progression. These cells have the ability to induce strong immunosuppressive effects within the tumor microenvironment (TME) and promote CRC development. Indeed, MDSCs have been shown to accumulate in both tumor-bearing mice and CRC patients, and may therefore become an obstacle for cancer immunotherapy. Consequently, numerous studies have focused on the characterization of MDSCs and their immunosuppressive capacity, as well as developing novel approaches to suppress MDSCs function with different approaches. Current therapeutic strategies that target MDSCs in CRC include inhibition of their recruitment and alteration of their function, alone or in combination with other therapies including chemotherapy, radiotherapy and immunotherapy. Herein, we summarize the recent roles and mechanisms of MDSCs in CRC progression. In addition, a brief review of MDSC-targeting approaches for potential CRC therapy is presented.

## Introduction

Colorectal cancer (CRC) remains the third most common cancer and the third leading cause of cancer-related deaths in males and females (1). Despite improvements in systemic treatments for advanced CRC in recent years, only 12–14% of patients with metastatic CRC survive for five years (2). Moreover, patients with advanced CRC develop resistance to chemotherapy, radiotherapy, immunotherapy and targeted drug therapy, which results in increasing challenges in treating CRC. Recently, different types of immune cells such as myeloid-derived suppressor cells (MDSCs), dendritic cells (DCs), tumor-associated macrophages (TAMs), natural killer (NK) cells, and regulatory T cells (Tregs) were shown to impact CRC progression (3, 4).

CRC patients’ responses to chemotherapy, radiation therapy, targeted drugs therapy and immunotherapy are affected by the tumor immune microenvironment (5, 6). Growing evidence has demonstrated that MDSCs accumulate and expand in the peripheral blood and tumor tissues, where they regulate host anti-tumor immune responses (7, 8). Once the MDSC population is expanded and activated in the immune system, it executes its numerous functions in tumor progression. MDSCs not only suppress anti-tumor immunity but also impede the efficacy of therapeutic agents for cancer treatment (9).

Immunosuppression is an important hallmark of most cancer growth and progression (10). In recent years, accumulating data have indicated that MDSCs, as one of the main immunosuppressive cell populations, are pivotal for cancer development (11). MDSCs represent a heterogeneous population of immature myeloid cells (IMCs) that fail to complete their differentiation into macrophages, DCs, or granulocytes (12). MDSCs consist of two large groups of cells: granulocytic or polymorphonuclear MDSCs (PMN-MDSCs) and monocytic MDSCs (M-MDSCs) (13). In general, the immunosuppressive function of MDSCs is regulated by multiple signaling pathways as well as interactions with several immune cell populations and mediators, which directly or indirectly suppress anti-tumor immunity and support cancer progression (7, 9). An increasing number of studies have focused on MDSCs, which are involved in regulation of the immune response in many types of cancer, but are poorly understood in CRC. It has been shown that different populations of MDSCs are observed in the peripheral blood and tumor tissue of CRC patients (14). A positive relationship between MDSCs and CRC progression including growth, metastasis, invasion, and angiogenesis has also been reported (15). Therapeutic agents targeting MDSCs have been proven to promote anti-tumor immunity and enhance the effects of immunotherapy against CRC. In this review, we discuss developments on the role of MDSCs in CRC: (1) MDSCs and their functional correlation with cancer, (2) MDSCs-mediated signaling pathways in CRC progression, and (3) MDSCs-targeting approaches for potential CRC treatment.

## Molecular Features and Suppressive Function of MDSCs

### Phenotypic Features

Under normal circumstances, IMCs do not have immunosuppressive functions and are believed to be constitutively present in healthy individuals. The generation of IMCs occurs in the bone marrow and is regulated by growth factors including granulocyte colony-stimulating factor (G-CSF), granulocyte macrophage colony-stimulating factor (GM-CSF), and macrophage colony-stimulating factor (M-CSF) (16, 17). During this process, IMCs migrate to the blood and various peripheral organs, where they differentiate into myeloid cells such as macrophages, neutrophils, and DCs. However, in pathological conditions including cancers, chronic infections and autoimmune diseases, IMC differentiation is impaired leading to an accumulation of MDSCs (18–20).

MDSCs are IMCs that expand during the growth and metastasis of malignant tumors and in inflammatory conditions. Their heterogeneity is tumor-dependent, and their phenotype and functions change with cancer progression (21). Recently, neutrophils were distinguished from MDSCs by the expression of the lectin-type oxidized LDL receptor 1 (LOX-1). LOX-1^+^ neutrophils have been shown to suppress T cells proliferation (22). These cells, also called tumor-associated neutrophils (TANs), were found in the tumor microenvironment (TME) and promote cancer cell migration and invasion (23). However, the main difference between MDSCs, neutrophils, and monocytes is their functions, with MDSCs having the potential to suppress immune activity.

There are two types of macrophages, namely, M1-like macrophage and M2-like macrophage. The macrophages found in the TME are known as TAMs and are predominantly M2 macrophages (24). TAMs are abundant in the microenvironment of CRC and are strong promoters of angiogenesis and lymphogenesis, thus contributing to tumor progression (25). Interferon gamma (IFN-*γ*), LPS, and GM-CSF induce polarization of M1 TAMs from monocytes, which are involved in antitumor immunity (26). In contrast, CSF-1, interleukin (IL)-4, IL-10, transforming growth factor beta (TGF)-*β*, and IL-13 contribute to M2 TAM polarization (27). M2 macrophages suppress cytotoxic T cell activities and attract Tregs, which promote tumor growth and immune escape (28). Furthermore, while M1 macrophages express CD64, suppressor of cytokine signaling 1 (SOCS1), indoleamine 2,3-dioxygenase (IDO) and chemokine (C-X-C motif) ligand 1 (CXCL1), M2 macrophages express mannose receptor C-type 1 (MRC1), CD23, and chemokine ligand 22 (CCL22) (29). Macrophages are highly plastic, and under certain physiological and pathological conditions, M1 macrophages can repolarize into M2 macrophages, and vice versa (30). However, the molecular mechanisms that regulate the macrophage polarization remain poorly understood.

Although TAMs and MDSCs are distinct cell types, they are not clearly distinguishable and have several characteristics in common. In the TME, cytokines and chemokines from tumor cells can influence normal myelopoiesis and increase the differentiation of M-MDSCs into PMN-MDSCs (31). M-MDSCs and inflammatory monocytes migrate to the tumor site *via* the CCL2/CCR2 pathways and differentiated into TAMs in response to various factors secreted by tumor cells (32). In addition, infiltrating MDSCs can also differentiate into TAMs through a combination of Toll-like receptor (TLR) and cytokine signaling (33, 34). MDSCs are phenotypically distinct from neutrophils, macrophages and monocytes. They can be divided into two major types based on their cell surface markers. In mice, PMN-MDSCs are defined as CD11b^+^Gr-1^+^Ly6G^high^Ly6C^low^ cells, whereas M-MDSCs are defined as CD11b^+^Gr-1^+^Ly6G^low^Ly6C^high^ cells. The frequencies of PMN-MDSCs and M-MDSC subsets differ between tumors and organs in tumor-bearing mice with PMN-MDSCs accounting for 70–80% of MDSCs and M-MDSCs representing 20–30% (35). In humans, PMN-MDSCs are HLA-DR-CD11b^+^CD14^−^CD33^+^ (CD15^+^ or CD66^+^) cells and M-MDSCs are HLA-DR^low/−^CD11b^+^CD14^+^CD15^−^ cells (36). Other cell surface molecules can also be used to identify other subsets of MDSCs, such as CD115, CD80, and CD124 (37, 38). Moreover, M-MDSCs express inducible nitric oxide synthase (iNOS) and generate nitric oxide (NO), while PMN-MDSCs produce reactive oxygen species (ROS) and arginase-1 (39).

In human peripheral blood, early-stage MDSCs (e-MDSCs), which are comprised of more immature progenitors than conventional MDSCs, are defined by Lin^−^ (including CD3, CD14, CD15, CD19, CD56) HLA-DR-CD33^+^. While e-MDSCs markers for murine cells have yet to be determined (36, 40–42), a subset of e-MDSCs with the phenotype of CD11b^+^Gr-1^−^F4/80^−^MHC-II^−^ has been described in IL-6 high-expressing 4T1 mice mammary carcinoma models (43). Human fibrocytic MDSCs (F-MDSCs), which can be differentiated from umbilical cord blood (UCB) precursors, have been identified as a new MDSCs subset with fibrocytic phenotypes and immunosuppressive functions. They are defined as CD11b^low^CD11c^low^CD33^+^IL-4Ra^+^ (44, 45). In addition, PMN-MDSCs and M-MDSCs are also phenotypically different from neutrophils. Compared to neutrophils, PMN-MDSCs have fewer granules and low expression of CD16 and CD62L (36, 41, 42, 46). The phenotypes and markers of MDSCs are shown in **Table 1**.

**Table 1:** Summary of commonly expressed markers in mice and human MDSCs.

MDSCs	PMN-MDSCs Markers	M-MDSCs Markers	e-MDSCs	F-MDSCs
Mice	CD11b^+^Gr-1^+^Ly6G^high^ Ly6C^low^	CD11b^+^Gr-1^+^Ly6G^low^Ly6C^high^	–	–
Human	HLA-DR-CD11b^+^ CD14^−^CD33^+^(CD15^+^/CD66^+^)	HLA-DR^low/−^CD11b^+^ CD14^+^ CD15^−^	Lin^−^(CD3/CD14/CD15/CD19/ CD56)HLA- DR-CD33^+^	CD11b^low^CD11c^low^CD33^+^IL-4Ra^+^

### Recruitment and Expansion of MDSCs

Cytokines can facilitate the recruitment and expansion of MDSCs in the TME. Tumor-induced factors, including prostaglandin E2 (PGE2), IL-6, IL-10, IL-1β, and transforming growth factor beta (TGF)-*β* have been shown to result in the recruitment and activation of MDSCs in the TME in malignant tumors (47). For example, IL-1β and IL-6 can induce the accumulation and activation of MDSCs at tumor sites (48–50). IL-1β not only promotes the accumulation of MDSCs but also induces expression of other molecules that are necessary for the expansion of MDSCs such as vascular endothelial growth factor (VEGF), IL-6 and GM-CSF (51). Other cytokines, such as IL-10 and TGF-*β* have the ability to generate MDSCs populations, as well as mediate their suppressive functions (52, 53). Thus, MDSCs are able to produce TGF-*β* and create a feedback loop that sustains their antitumor immunity. Furthermore, IL-17, which is secreted by Th17 cells, is overexpressed in cancer cells and promote MDSCs translocation from the periphery to the tumor sites (54).

Chemokines also influence MDSCs expansion and activation. Some of them, such as chemokine ligand 2 (CCL2) can interact with its corresponding receptor C-C chemokine receptor type 2 (CCR2) to promote chemotaxis to areas of inflammation (55, 56). IL-8 is released by cancer cells and binds to G protein-coupled receptors C-X-C motif chemokine receptor 1 and 2 (CXCR1 and CXCR2) on MDSCs (57). Moreover, the CCL3/CCR5 axis has been reported to induce the maintenance of immunosuppressive myeloid cells in tumor areas (55).

Hypoxia is commonly found in the TME and is recognized as an important factor that mediates MDSCs expansion. It has been shown that hypoxia inducible factor 1 alpha (HIF-1*α*) can induce the expression of ectonucleoside triphosphate diphosphohydrolase 2 (ENTPD2), an ectoenzyme on MDSCs, resulting in MDSCs expansion in the TME (40). In addition, hypoxia can upregulate VEGF and functional molecule expression and lead to MDSCs accumulation in both mice and patients with lung cancer (58, 59). This process is mediated by the VEGF receptor, which is expressed on MDSCs (58).

### MDSCs Immunosuppressive Mechanism

Notably, one of the features of MDSCs in TME is the immune suppressive function. MDSCs suppress the activity of immune cells through multiple mechanisms, including the generation of reactive oxygen and nitrogen species (RONS), the degradation of L-arginine, the production of immunosuppressive cytokines such as IL-10 and TGF-*β*, the inhibition of T cells, and the induction of other immunosuppressive cells (41, 60). Firstly, MDSCs can regulate anti-tumor immune responses through the production of RONS including NO and ROS (61, 62). MDSCs require activation of signal transducer and activator of transcription 3 (STAT3) and increase nicotinamide adenine dinucleotide phosphate (NADPH) oxidase activity to produce ROS (13, 41). However, NADPH oxidase may also synthesize reactive nitrogen species (RNS) like NO by metabolizing L-arginine (63). ROS are also activated *via* the STAT3 transcription factor and are associated with the metabolism of L-arginine (52, 64). Taken together, these data suggest that production of ROS, NO, and RNS are dependent on L-arginine metabolism. Furthermore, these factors can suppress T cell populations, thus rendering them incapable of facilitating an anti-cancer response (65). In the TME, S100A8/A9 has been shown to activate the production of ROS in a STAT3-dependent manner. This leads to nitration of the T cell receptor-alpha-beta (TCR*αβ*) chain, resulting in T cells that lack the ability to interact with the peptide antigen bound to the major histocompatibility complex class II (MHC-II) and are therefore unable to initiate an anti-cancer response (51). Similarly, iNOS released by MDSCs is an additional mechanism responsible for inducing oxidative stress in the TME. NO produced by iNOS can suppress the T cells’ response and induce T cell apoptosis *via* various mechanisms, including the inhibition of Janus kinase 3(JAK3), STAT5, and MHC-II expression (11, 13). Synergistically, S100A8/A9 also increases the production of iNOS through activation of STAT1 (66).

Secondly, MDSCs can suppress T cell activation and proliferation by depleting essential amino acids. MDSCs increase arginase-1 activity and induce T cell suppression *via* the depletion of L-arginine (11, 13, 67). The lack of L-arginine suppresses proliferation of activated T cells and decreases the expression of the T cell receptor-zeta (TCR-ζ) chain (68). As a result, arginase-1 leading to depletion of L-arginine in the TME suppresses the ability of the T cells to exert their anti-tumor functions (69). Indeed, MDSCs have the ability to inhibit T cell proliferation by regulating the G0/G1 phase of their cell cycle (70). Expression of IDO by MDSCs can also suppress T cell proliferation by decreasing tryptophan levels and producing cytotoxic metabolites (71). Furthermore, it was reported that chronic psychological stress can also lead to MDSCs accumulation in the bone marrow of Balb/c mice where they inhibit T cells proliferation (72).

Other mechanisms that result in MDSCs-induced T cell apoptosis have been described. For example, MDSCs can decrease expression level of B-cell lymphoma 2 (Bcl-2) expression and increase FAS (CD95 ligand) expression in T cells. Furthermore, MDSCs express galectin-9, which binds to T-cell immunoglobulin domain and mucin domain 3 (TIM3), an inhibitory surface molecule on lymphocytes, leading to decreased T cell viability (73, 74). Interestingly, different subtypes of MDSCs utilize different mechanisms to mediate immunosuppressive functions in the TME. M-MDSCs produce high levels of NO and immunosuppressive cytokines such as IL-10, which suppress both antigen-specific and non-specific T-cell responses (55). In contrast, PMN-MDSCs suppress T-cell responses by generating ROS based on an antigen-specific approach (75, 76).

**Figure 1:**
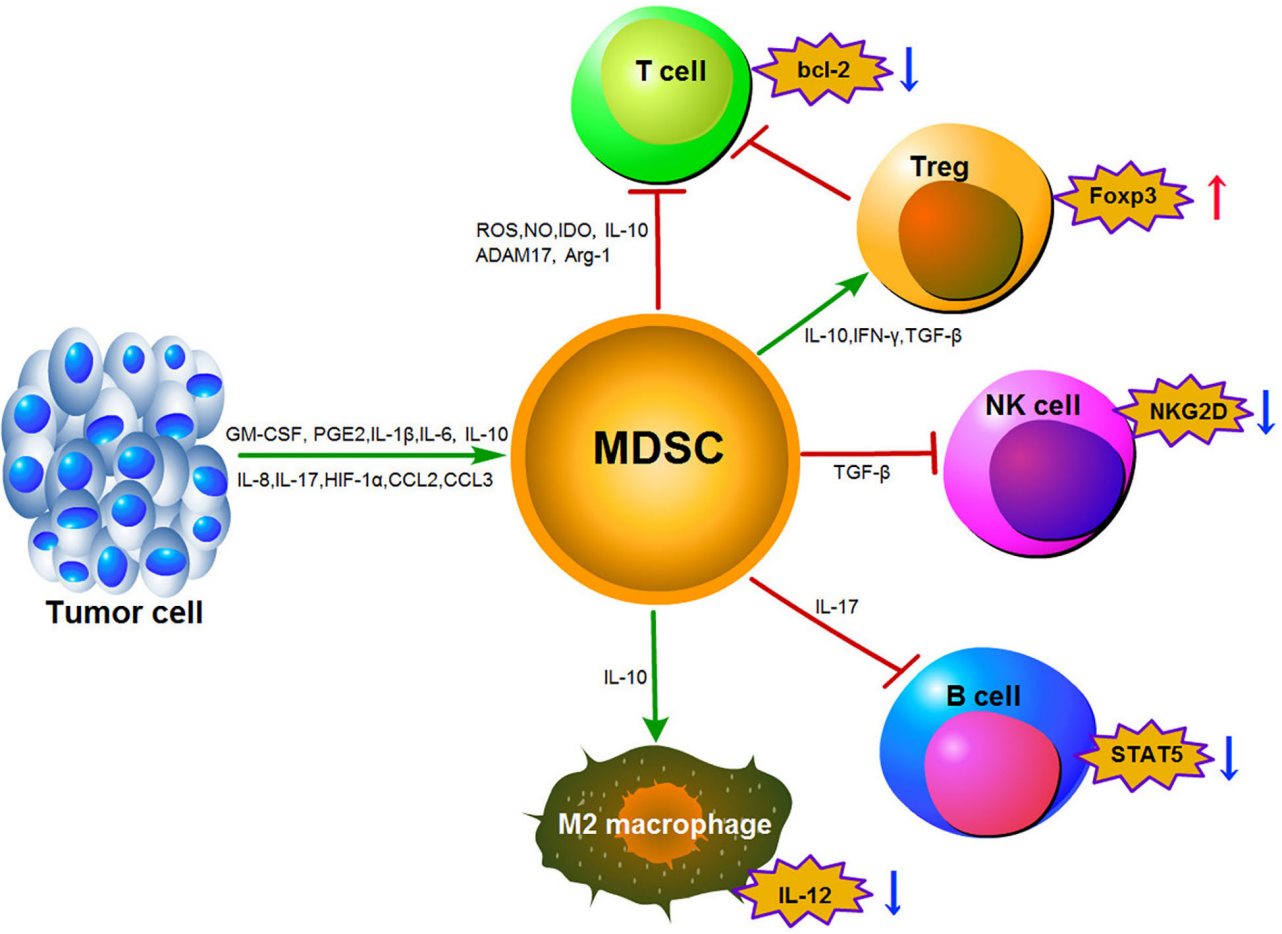
Main mechanisms of immunosuppression function mediated by MDSCs in the tumor microenvironment.

Thirdly, MDSCs-mediated lymphocyte trafficking and viability are restricted. MDSCs can suppress T cell movement to the lymph nodes *via* down-regulation of L-selectin (CD62L) on the surface of T cells by increasing expression of disintegrin and metalloproteinase domain17 (ADAM17). MDSCs can also interrupt the migration of CD8+ T cells to tumor sites by peroxynitrite modification of CCL2 (77, 78). Finally, MDSCs can promote the induction of other immunosuppressive cells. MDSCs have been shown to induce the generation of FoxP3^+^ Treg cells *in vivo* through the production of IFN-*γ*, TGF-*β* and IL-10. This effect is independent of NO production (38). Furthermore, the CCR5 ligands CCL3, CCL4, and CCL5 were shown to promote CCR5^+^ Treg cell recruitment in mouse models of melanoma (74). It has been reported that CD14^+^HLA-DR^–/low^ M-MDSCs could induce CD4^+^CD25^+^Foxp^3+^Treg cells when co-cultured with autologous T cells in hepatocellular carcinoma (HCC) patients (79). Moreover, F-MDSCs can inhibit T cell proliferation by releasing IDO and promoting the expansion of Tregs (80). With the exception of Tregs stimulation, MDSCs can also reverse macrophages to an M2-like phenotype with low IL-12 production, thereby promoting tumor growth (81). In addition, MDSCs impair NK cell function and cytotoxicity by suppressing the production of IFN-*γ* from NK cells and decreasing the expression of natural killer group 2 member D (NKG2D) (82). Induction of MDSCs in a tumor-bearing mouse model of lung cancer can lead to impairment of B cell differentiation and function though an IL-7 and STAT5-dependent manner (83). The MDSCs immunosuppressive mechanisms described above are outlined in **Figure 1**.

## Roles of MDSCs in Colorectal Cancer

### Prognostic Values of MDSCs in CRC

MDSCs play an important role in the immunosuppressive mechanism associated with CRC progression. Several studies have demonstrated that MDSCs in the peripheral blood and tumor tissues are associated with tumor stage, histological grade of cancer and lymph node metastases in CRC (84, 85). It has been reported that the peripheral blood of CRC patients contains a significantly increased percentage and absolute number of CD33^+^CD11b^+^HLA-DR^−/low^ MDSCs compared with healthy donors. This increasement was closely correlated with clinical cancer stage and tumor metastasis but not primary tumor size. Interestingly, radical operation can significantly decrease the level of circulating MDSCs in CRC patients (76, 85). Moreover, the proportion of PMN-MDSCs and immature MDSCs (I-MDSCs) was found to be higher in the tumor tissues of CRC patients compared to tumor-adjacent tissues (86).

In a recent study, CD33^+^ MDSCs and Yes-associated protein 1 (YAP1) were identified as predictors for the prognosis of CRC patients. This study demonstrated that CD33^+^MDSCs numbers and YAP1 expression levels were increased in tumor tissues compared with those of tumor-adjacent tissues from the same CRC patients (87). Furthermore, CD33^+^CD11b^+^HLA-DR^−/low^ myeloid cells were shown to be expanded in the peripheral blood of CRC patients, with the number of circulating MDSCs positively correlating with poor prognosis and low survival rates (88). In addition, a lower lymphocyte-to-monocyte ratio (LMR) was associated with poor prognosis in CRC patients, who were found to have higher levels of circulating MDSCs (89). Unresectable metastatic CRC patients with high M-MDSCs levels in their peripheral blood were also shown to have a significantly shorter progression-free survival (90). Interestingly, it was proven that although Tregs, Th17, and PMN-MDSCs were significantly increased in metastatic CRC, only high levels of PMN-MDSCs were associated with a poor prognosis for CRC patients (91).

### Signaling Pathways for MDSCs-Mediated Functions in CRC

The immunosuppressive function of MDSCs relies on the activation of different intracellular signaling pathways. Many studies indicate that MDSCs-associated signaling pathways are involved in CRC development. KRAS mutations, for example, are frequently observed in human CRC and correlate positively with tumor aggressiveness and metastasis (92–94). KRAS-mediated repression of interferon regulatory factor 2 (IRF2) was associated with high expression of CXCL3, which led to MDSCs migration to the TME through binding to CXCR2 on the MDSCs (95). Receptor-interacting protein kinase 3 (RIPK3) is essential for mucosal repair in CRC. It has been reported that reduction of RIPK3 in CRC cells induces expansion of MDSCs and increases cyclooxygenase-2 (COX-2) expression, which then catalyze PGE2 and enhance MDSCs function (96). Similarly, RIPK3 signaling in an I-MDSCs subset promoted intestinal tumor development in MC38 cell tumors by expanding IL17-producing T cells (97).

It has been suggested that down-regulation of mucin 1 (MUC1) in hematopoietic cells increases MDSCs expansion in inflammatory bowel disease (IBD) leading to the development of CRC (98). Moreover, MUC13 promotes colitis-associated colorectal tumors development through the *β*-catenin signaling pathway and increases MDSCs expansion in the TME (99). In addition, MDSCs can increase the expression levels of ROS and NO, which may result in DNA damage and promote tumor progression in CRC (100). Previous studies have also demonstrated that CRC cells secrete VEGF-A, which leads to TAMs induction and subsequent production of chemokine (C-X-C motif) ligand 1 (CXCL1) in the primary tumor. Increased CXCL1 in liver tissue was shown to recruit CXCR2-positive MDSCs to form a premetastatic niche in CRC (101). In addition, overexpression of CXCR4 has been found to play a crucial role in the invasion of CRC, as well as promoting the epithelial–mesenchymal transition (EMT) and infiltration of MDSCs in colonic tissue, accelerating colitis-associated and Apc mutation-driven colorectal tumorigenesis (102). Recently, Varun Sasidharan Nair et al. reported some genes associated with histone deacetylases (HDAC) activation, DNA methylation, Wnt and IL-6 signaling pathways are upregulated in CRC tumor infiltrating I-MDSCs, and propose that they could be exploited as potential targets for CRC therapy (86).

The JAK/STAT pathway is considered to be a major player in mediating immunosuppression (103, 104). MDSCs isolated from the spleen of CT26 cell-bearing mice exhibited inhibition of phosphorylation of STAT1 (p-STAT1) in response to IFN-α or IFN-*γ* (105). However, another study demonstrated that IFN-*γ* is not a key regulator of MDSCs and that targeting it would be unlikely to alter MDSCs accumulation or function in tumor-bearing mice (106). It has been reported that IL-6 activates expansion of MDSCs *via* the JAK2/STAT3/NF-*κ*B signaling pathway, resulting in AOM/DSS-induced colon tumor development in G protein subunit alpha i1 (GNAI1) and GNAI3 (GNAI1;3) double-knockout (DKO) mice (107). It has also been shown that protease-activated receptor 2 (PAR2) deficiency significantly promotes tumor development in the AOM/DSS-induced colitis-associated colon cancer model through accumulation of MDSCs and enhancement of their immunosuppressive activity *via* STAT3-mediated ROS production (108). Previous studies have demonstrated that G-CSF could promote MDSCs’ survival and activation through the STAT3 signaling pathway in a mouse colitis-associated cancer model (109). Additionally, CCL2 was initially characterized as a cytokine that was found to be increased in CRC tissues and reported to enhance PMN-MDSCs’ function in a STAT3-mediated manner (110). Xu et al. demonstrated that sphingosine-1-phosphate receptor 1 (S1PR1) and STAT3 are elevated in human CRC tissues and propose that they recruit MDSCs through the S1PR1–STAT3–IL-6 axis to promote tumor growth and liver metastasis niche (111). In addition, STAT6 appears to promote expansion of MDSCs and contributes to intestinal tumor progression in ApcMin/+ mice (112). Finally, S100A8/A9 is another pro-inflammatory molecular that activates the STAT3 signaling pathway, which is responsible for maintaining the MDSCs suppressive function (113).

Of significance, a highly hypoxic environment leads to the accumulation and activation of MDSCs in CRC development. Hypoxia within the TME is associated with increased V-domain Ig suppressor of T-cell activation (VISTA) expression, which promotes MDSCs function. VISTA is highly expressed in the CRC microenvironment, while both VISTA and HIF-1*α* activity were found to be increased in a cohort of CRC patients (114). Notably, malignant tumors can potentially recruit MDSCs from the bone marrow by releasing exosomes. Previous studies have shown that hypoxia can induce MDSCs to secrete more exosomes in a HIF-1*α* dependent manner (115). The exosomal contents can reprogram the target cell and increase mobility of MDSCs to the tumor sites. Inhibition of S100A9 was found to suppress the susceptibility of mice to AOM/DSS-induced colitis-associated colon cancer (116).

### Targeting MDSCs in CRC Therapy

**Figure 2:**
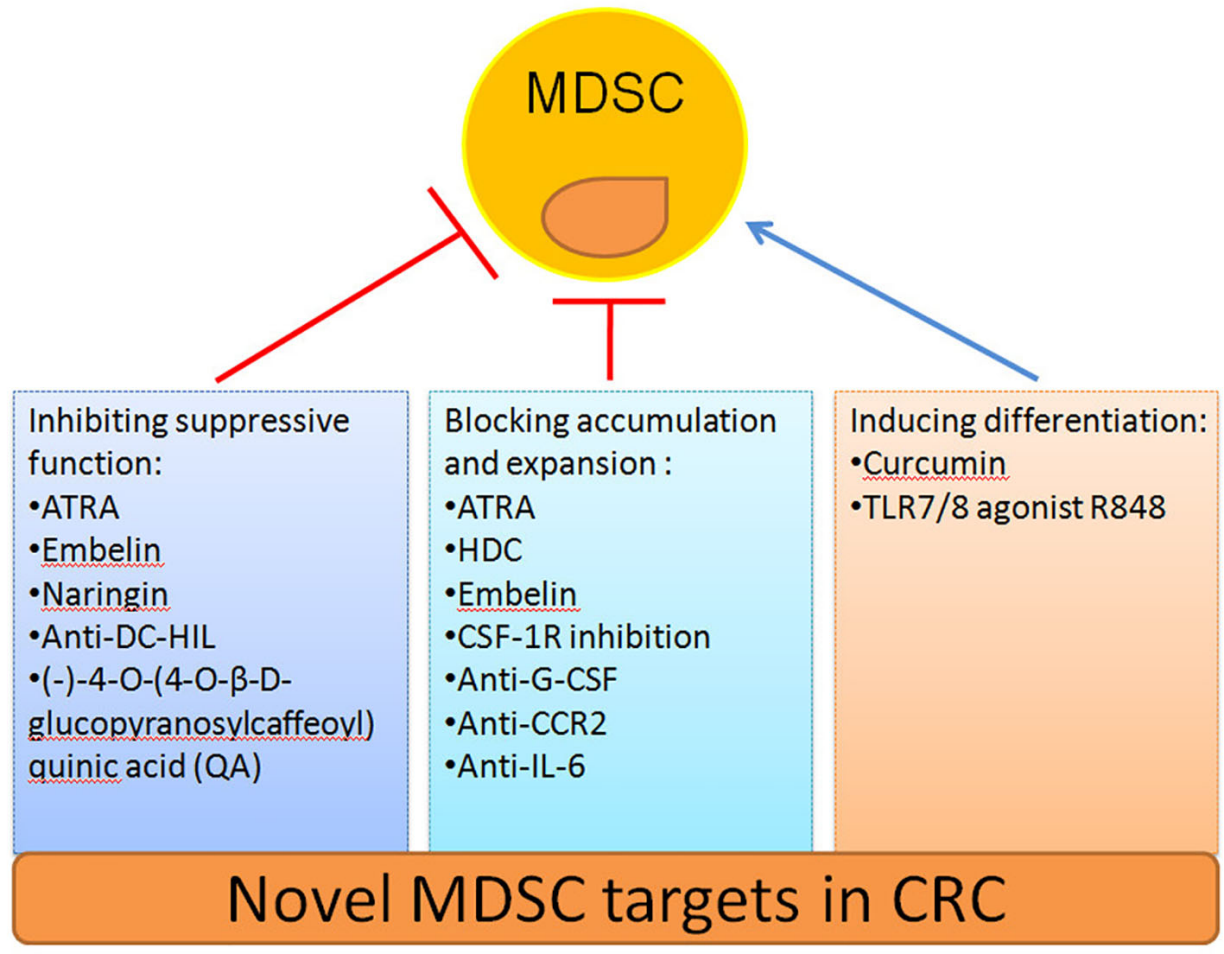
Novel strategies to target MDSCs in CRC.

The safety and efficacy of using MDSCs inhibition as a CRC therapy have been evaluated in an increasing number of studies (**Table 2**). Here we summarize novel preclinical approaches targeting MDSCs in CRC (**Figure 2**). Current treatments aim to deplete MDSCs, inhibit their immunosuppressive function, and block their expansion to the tumor site (128). Several studies have proved that depletion of the number of MDSCs and inhibition of their function in tumor tissue are an important strategy for CRC therapy. For example, targeting MDSCs with all-trans-retinoic acid (ATRA) has been shown to decrease their number and suppress their function in tumor bearing mice (117). Consequently, ATRA may consider being a novel immunotherapeutic protocols to target CRC in the future. Similarly, histamine dihydrochloride (HDC), a NADPH oxidase 2 (NOX2) inhibitor, has also been shown to inhibit tumor progression by reducing the accumulation of tumor MDSCs in MC-38 cell-bearing mice (118). Embelin (2,5-dihydroxy-3-undecyl-1,4-benzoquinone) is a non-peptidic small molecule inhibitor of X-linked inhibitor of apoptosis protein (XIAP). Wu et al. found that embelin can significantly reduce the accumulation number of MDSCs in the peripheral lymphoid organ and tumor tissue, and impair the immunosuppressive function of MDSCs by reducing the production of ROS and arginase-1 level in colitis-associated tumorgenesis (119). Naringin (4′,5,7-trihydroxyavanone-7-rhamnoglucoside), a major flavanone glycoside that occurs naturally in citrus fruits, inhibits the severity of colitis and CRC development through regulation of the MDSCs’ immunosuppressive function *via* the NF-*κ*B/IL-6/STAT3 axis in colorectal tissues (120).

**Table 2:** Summary of preclinical studies analyzing the role of MDSCs in CRC and therapeutic agents.

Mouse models	Cells lines	Therapeutic agents	Targets	Ref.
C57/BL6 mice	MC38 cells	all-trans-retinoic acid (ATRA)	–	(117)
C57/BL6 mice and Nox2-KO mice	MC38 cells	Histamine dihydrochloride (HDC)	NOX2	(118)
C57BL/6 mice	–	Embelin	XIAP	(119)
C57BL/6 mice	–	Naringin	–	(120)
Balb/c mice	CT26 cells	CSF-1R kinase inhibitors (PLX647 and PLX5622)	CSF-1R	(121)
C57/BL6 mice and SCID mice	–	anti-G-CSF mAb	G-CSF	(109)
C57BL/6 mice	MC38 cells	anti-DC-HIL mAb	DC-HIL	(122)
Balb/c mice	CT26 cells	Curcumin	–	(123)
Balb/c mice	CT26 cells	TLR7/8 agonist, R848	TLR7/8	(124)
C57BL/6 mice,CCR2^−/−^ and CCL2^−/−^ mice	MC38 cells	anti-CCR2 mAb	CCR2	(125)
Balb/c mice and C57BL/6 mice	CT26 cells and MC38 cells	anti-IL-6 mAb	IL-6	(126)
Balb/c mice	CT26 cells	(-)-4-O- (4-O-β-D-glucopyranosylcaffeoyl) quinic acid (QA)	PI3Kδ/γ	(127)

Recruitment of tumor MDSCs is dependent on the receptor tyrosine kinase CSF-1R. Thus, inhibition of CSF-1R signaling can significantly block the number of tumor-infiltrating MDSCs number and enhance anti-tumor T cells responses in tumor bearing mice (121). In addition, blocking the immunosuppressive function of MDSCs can be achieved by targeting anti-G-CSF monoclonal antibody. Treatment with an anti-G-CSF monoclonal antibody reduces MDSCs accumulation and decreases the migration, proliferation, and functional maintenance of MDSCs and could therefore become a potential therapeutic agent for colitis-associated cancer (109). Previous results have indicated that DC-HIL^+^MDSCs are expanded in the blood of metastatic CRC patients. Since, anti-DC-HIL mAb treatment can suppress the function of MDSCs isolated from treated mice, functionally blocking DC-HIL on MDSCs could also be potentially beneficial in the treatment of metastatic CRC (122).

Other treatments include the induction of MDSCs differentiation alone or in combination with radiotherapy, chemotherapy, surgery or other kinds of immunotherapy to target CRC (129). Curcumin has been shown to inhibit the expansion of MDSCs, activate STAT3 and NF-*κ*B in MDSCs, and polarize MDSCs toward a M1-like phenotype in CT26 cell-bearing mice (123). Recently, the TLR7/8 agonist R848, as a new immunologic adjuvant, was found to reverse the functional orientation of MDSCs towards M1 macrophages, suggesting that R848 may be a potential immunologic adjuvant in chemotherapy for oxaliplatin-resistant CRC (124). It has been reported that treatment with anti-CCR2 antibody can alleviate radiation-induced MDSCs infiltration in CRC tumor tissues by activation of the STING pathway (125). Hence, anti-CCR2 antibody treatment may improve radiotherapy for advanced CRC patients. A previous study indicated that IL-6 induces strong immunosuppression in the CRC microenvironment by recruiting MDSCs and impairing T cells infiltration. Interestingly, an anti-IL-6 and anti-PD-L1 combination treatment prolonged tumor-bearing mouse survival, providing a novel strategy to overcome anti-PD-L1 resistance in CRC (126). In addition, blocking the immunosuppressive function of MDSCs can also be achieved by targeting phosphatidylinositol 3-kinase (PI3K)*δ/γ*. Hence, (-)-4-O-(4-O-β-D-glucopyranosylcaffeoyl) quinic acid (QA), a selective small molecule inhibitor of PI3K*δ*/*γ*, has the ability to reshape the tumor immune microenvironment and promote responses to anti-PD-1 treatment in a colon tumor model (127).

While there is an abundance of preclinical data supporting the theory that suppression of MDSCs could be a beneficial therapeutic tool. Several clinical studies have also indicated that inhibition of MDSCs is beneficial to CRC patients. Metastatic CRC patients treated with a first-line combination regimen of 5-FU, oxaliplatin, and bevacizumab (FOLFOX-bevacizumab) were associated with a better survival outcome. Furthermore, the FOLFOX-bevacizumab treatment was found to decrease the PMN-MDSC population in metastatic CRC patients (91). While docosahexaenoic acid (DHA) has been shown to inhibit caspase-1 activity in 5-fluorouracil (5-FU) treated MDSCs, a negative relationship between the DHA content in plasma and the induction of caspase-1 activity in MDSCs of CRC patients treated with 5-FU-based chemotherapy has been reported (130). Thus, these data provide new insights into the regulation of DHA and its potential benefit in 5-FU-based chemotherapy for CRC patients. Previous studies demonstrated that CD38 is a transmembrane receptor–ectoenzyme expressed by MDSCs in esophageal cancer and multiple myeloma (131, 132). Interestingly, a significant expansion of CD38^+^M-MDSCs were observed in PBMCs of CRC patients when compared with healthy donors, and CD38^+^M-MDSCs frequencies were significantly higher in CRC patients who had previously received any form of cancer treatment (surgery, chemotherapy or radiotherapy, targeted therapy, or a combination of these methods) when compared with treatment-naive patients (133). This study supported a method to target M-MDSCs with an anti-CD38 monoclonal antibody could be a valuable therapeutic tool for the treatment of metastatic CRC patients.

### Immune Checkpoints

**Figure 3:**
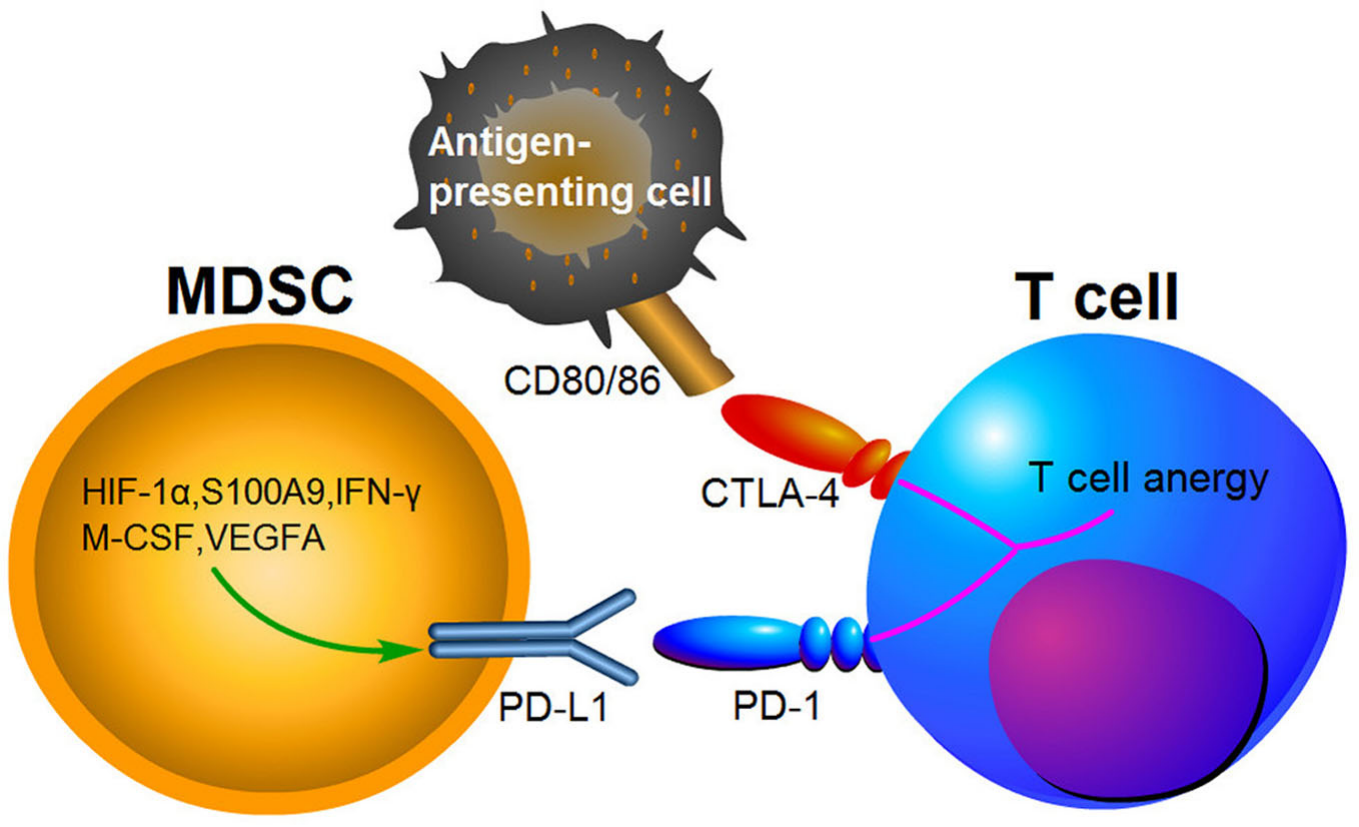
The expression of PD-L1 in MDSCs causing T cell anergy *via* binding to its respective receptor.

Growing evidence suggests that the immunosuppressive microenvironments in tumors result from the activation of MDSCs, PD-1/PD-L1 and cytotoxic T lymphocyte-associated antigen-4(CTLA-4) pathways (134). PD-1 has been found to bind to its ligand PD-L1 and then induce T cell anergy and apoptosis (135). CTLA-4 is another receptor expressed on the surface of T cells, which inhibits T cells activities by competing with CD28 to bind to the two T cell activation antigens, CD80 and CD86 (136). PD-1 and CTLA-4 are immune checkpoint proteins expressed on activated T cells (**Figure 3**). Blocking PD-L1 or CTLA-4 signaling has been shown to be beneficial for cancer patient survival. MDSCs express high levels of PD-L1, and this upregulation of PD-L1 has been associated with expression of S100A9 and HIF-1*α* (69, 137). It has been indicated that PD-L1 expression on MDSCs is increased in CRC patients and colon tumor bearing mice, suggesting that it may be a potent mediator of immunosuppression function (138, 139). Furthermore, PD-L1^+^MDSCs were significantly decreased after neutralization of IFN-*γ* in the TME (139). Interestingly, PD-L1^+^MDSCs are significantly increased in HCC patients, while M-CSF and VEGFA have been shown to induce PD-L1^+^MDSCs *in vitro* (140). Compared to responding patients, PMN-MDSCs also expressed high levels of PD-L1 in non-responding melanoma patients treated by ipilimumab (141).

Several studies found that MDSCs and other molecules such as PD-L1 and CTLA-4 in tumor tissues are sensitive predictive markers for patients’ response to chemoradiotherapy for rectal cancer patients (142). In the latest study, it was demonstrated that blockade of CXCR2 on MDSCs can overcome resistance to anti-PD-1 therapy in CRC-expressing oncogenic KRAS (95). Similarly, MDSCs elimination can reverse resistance to anti-PD-L1 or combination of normo-fractionated radiotherapy plus immunotherapy in CRC (143). Moreover, HDC can reduce the accumulation of intratumoral MDSCs in colon tumor-bearing mice and improve the anti-tumor efficacy of the PD-1/PD-L1 checkpoint blockade (118). Interestingly, IL-6 blockade was also reported to reverse the anti-PD-L1 resistance and inhibit CRC growth by reducing the number of MDSCs (126). MUC13-deficient mice have fewer MDSCs and are sensitive to anti-PD-L1 therapy, suggesting that MUC13 may be useful for in the treatment of colitis-associated cancer (99). Additionally, it has been revealed that treatment using an anti-KIT monoclonal antibody in a mouse colon cancer model enhanced the anti-tumor activity of anti-CTLA-4 and anti-PD-1 therapy by selectively reducing the MDSCs population (144).

Several studies have shown that the increasing number of TAMs in tumor correlates with favorable 5-year overall survival (OS) for CRC patients (145, 146). Increased M2 macrophage numbers in the TME promote the initiation and growth of tumor. However, few strategies are currently available to modulate TAMs by repolarizing the M2 macrophages to become M1 macrophages. A recent study demonstrated that PD-L1^+^ T cells can engage PD-1^+^ macrophages, inducing an alternative M2-like program, which have effects on adaptive antitumor immunity (147). In addition, Wang et al. showed that CD30L deficiency promote the accumulation of MDSCs, increase the expression of PD-L1 on MDSCs and TAMs, and enhance immunosuppressive function in an AOM/DSS-induced CAC model, suggesting that CD30L/CD30 signaling could be a potential candidate target for immunotherapy in CAC (148).

## Conclusions

In conclusion, numerous studies have documented the important role of immunosuppressive MDSCs in CRC development in mice and cancer patients. During CRC progression, MDSCs-mediated immunosuppressive activity is regulated by many different signaling pathways. MDSCs promote CRC progression by increasing cell proliferation, cancer stemness, enhancing tumor invasiveness and metastasis. Given that the mechanisms controlling expansion and activation of MDSCs in tumor tissues or in the peripheral blood are distinct, it is difficult to devise a therapeutic approach to reduce their numbers or arrest their function. Furthermore, monotherapies targeting MDSCs have shown promising but limited efficacy. Thus, it is important to elucidate novel mechanisms involving different stromal components and myeloid cells such as cancer associated fibroblasts (CAFs), TAMs and neutrophils. Further studies are required to strengthen the knowledge about MDSCs and to better understand the effects in combination with other therapies involving different immunotherapeutic approaches for CRC therapy.

## Author Contributions

KY drafted the manuscript. XX and KR discussed and revised the manuscript. TW and SW designed the study and provided critical suggestions. All authors contributed to the article and approved the submitted version.

## Funding

This study was conducted with support from the Natural Science Foundation of Jiangsu (Grant No. BK20190242 and BK20170563), Jiangsu Province’s Key Medical Talents Program (Grant No.ZDRCB2016018), Research Project of Jiangsu Commission of Health (Grant No. K2019019).

## Conflict of Interest

The authors declare that the research was conducted in the absence of any commercial or financial relationships that could be construed as a potential conflict of interest.

## Abbreviations

DCs, Dendritic cells; TAMs, tumor-associated macrophages; NK, natural killer; Tregs, regulatory T cells; UCB, umbilical cord blood; MDSCs, myeloid-derived suppressor cells; IMCs, immature myeloid cells; PMN-MDSCs, polymorphonuclear MDSCs; M-MDSCs, monocytic MDSCs; e-MDSCs, early-stage MDSCs; PBMCs, peripheral blood mononuclear cells; F-MDSCs, fibrocytic MDSCs; PGE2, prostaglandin E2; TGF-*β*, transforming growth factor beta; HIF-1*α*, hypoxia-inducible factor-1α; CRC, colorectal cancer; CCR2, C-C chemokine receptor type 2; iNOS, inducible nitric oxide synthase; CCL2, chemokine ligand 2; CXCR, C-X-C motif chemokine receptor; ENTPD2, ectonucleoside triphosphate diphosphohydrolase 2; RONS, reactive oxygen and nitrogen species; RNS, reactive nitrogen species; G-CSF, granulocyte colony-stimulating factor, GM-CSF, granulocytes macrophage colony-stimulating factor; M-CSF, macrophage colony-stimulating factor; STAT3, signal transducer and activator of transcription 3; NADPH, nicotinamide adenine dinucleotide phosphate; TME, tumor microenvironment; NF-*κ*B, nuclear factor-*κ*B; NO, nitric oxide; ROS, reactive oxygen species; VEGF, vascular endothelial growth factor; JAK3, Janus kinase 3; MHC, major histocompatibility complex; IDO, indoleamine 2,3dioxygenase; Bcl-2, B-cell lymphoma 2; TIM3, T-cell immunoglobulin domain and mucin domain 3; ADAM17, disintegrin and metalloproteinase domain17; IFN-γ, interferon gamma; HCC, hepatocellular carcinoma; IRF2, interferon regulatory factor 2; RIPK3, receptor-interacting protein kinase 3; COX-2, cyclooxygenase-2; IBD, inflammatory bowel disease; VISTA, V-domain Ig suppressor of T-cell activation; GNAI1, G protein subunit alpha i1; PAR2, protease-activated receptor 2; S1PR1, sphingosine-1-phosphate receptor 1; ATRA, all-trans-retinoic acid; HDC, histamine dihydrochloride; XIAP, X-linked inhibitor of apoptosis protein; PI3K, phosphatidylinositol 3-kinase; CTLA-4, cytotoxic T lymphocyte-associated antigen-4.
